# Childhood Traumatic Brain Injury and the Associations With Risk Behavior in Adolescence and Young Adulthood: A Systematic Review

**DOI:** 10.1097/HTR.0000000000000289

**Published:** 2017-01-13

**Authors:** Eleanor Kennedy, Miriam Cohen, Marcus Munafò

**Affiliations:** MRC Integrative Epidemiology Unit at the University of Bristol, UK Centre for Tobacco and Alcohol Studies, School of Experimental Psychology, University of Bristol, United Kingdom (Ms Kennedy and Dr Munafò); and College of Life and Environmental Sciences, University of Exeter, United Kingdom (Ms Cohen).

**Keywords:** antisocial behavior, child, conduct disorder, head injury, pediatric, risk behavior, substance use, systematic review, traumatic brain injury

## Abstract

**Objective:**

To systematically review the evidence that childhood traumatic brain injury (TBI) is associated with risk behavior in adolescence and young adulthood. Risk behavior included one or more of the following: use of substances, including alcohol, tobacco, and illicit substances; involvement in criminal behavior; and behavioral issues with conduct.

**Methods:**

A literature search was conducted using these terms: child, pediatric, traumatic brain injury, head injury, adolescent, psychosocial, antisocial, conduct, substance use. Studies describing original research were included if they reported outcomes over the age of 13 years in participants who sustained a TBI between birth and age 13 years.

**Results:**

Six journal articles were reviewed based on 4 separate studies. Three articles indicated a relationship between childhood TBI and increased problematic substance use in adolescence and young adulthood. Three articles supported an association between childhood TBI and later externalizing behavior; however, 2 articles did not support this link.

**Conclusion:**

More research is warranted to explore the association between childhood TBI and later risk behavior as the relationship is not currently understood. Future research should build on existing longitudinal research with continued use of medical records for identifying TBI and inclusion of a non-brain-related trauma group to control for general injury effects.

## Abstract 2

Supplemental Digital Content is Available in the Text.

THE WORLD HEALTH ORGANIZATION classifies traumatic brain injury (TBI) as the leading cause of death and disability among children and young adults globally.^1^ Yates and colleagues^2^ estimate the prevalence rate for moderate-to-severe TBI in children younger than 5 years to be approximately 120 per 100 000 for those living in urban areas.

TBI is associated with cognitive, behavioral, and emotional problems.^3^ Although a peak in recovery of function within the first 6 to 12 months following a childhood TBI is often reported,^4^ longer term effects of a childhood TBI may not become apparent until later developmental stages, when more complex demands are placed on an individual.^5^ Adolescence is a time of increased demand as an individual transitions to relative independence, and enhanced social cognitive skills are required to navigate increasingly intricate and intimate relationships.^6^ An increase in risk-taking behavior is also typically seen in adolescence. Steinberg^7^ argues that the heightened salience of peer relations in adolescence is key to the increased risk-taking behavior seen at this age. Chein and colleagues^8^ reported that the presence of peers increased the number of risks taken by adolescents in a simulation driving task. In a functional magnetic resonance imaging task, adolescents being observed by peers had greater activation of reward-related brain regions, including the ventral striatum and the orbitofrontal cortex, than the 2 older age groups.^8^

Hessen and colleagues^9^ carried out a follow-up study in patients admitted to hospital for a mild TBI; 45 people who were injured before age 15 years and 74 injured after age 15 years completed a comprehensive assessment of neuropsychological function 23 years after their index injury. The authors found mean test scores within the normal range for the total sample combined across age groups. However, in the group injured during childhood, mild TBI with posttraumatic amnesia lasting over 30 minutes or posttraumatic amnesia of over 30 minutes in combination with a pathological electroencephalogram within 24 hours was strongly predictive of poor neuropsychological outcome. This was not the case for adults with the same diagnostic variables, which the authors suggested was indicative of greater vulnerability in children to the long-term consequences of complicated mild TBI than adults.

Associations between TBI events in childhood and later risk behavior have been reported in previous research. Findings from the Christchurch Health and Development Study birth cohort suggest that participants who had a TBI between birth and age 5 years were more likely to report alcohol and drug dependence and also more likely to have been involved in violent offenses than participants with no TBI history.^10^ Other research on the same cohort indicated higher parent and teacher ratings of hyperactivity/inattention and conduct disorder for participants injured between the ages of 0 and 10 years 11 and increased likelihood of conduct disorder and substance abuse in participants who had a TBI event between birth and age 5 years^12^; however, both results were only observed for participants whose injury resulted in an inpatient hospital stay.

Tonks and colleagues^13^ found higher parent and teacher ratings of social difficulties at age 10 to 16 years in participants who had experienced a TBI approximately 4 years previous, and also for participants aged 8 to 10 years for whom a TBI event occurred between birth and age 5 years. When compared with orthopedic injury controls, 8- to 13-year-old participants with a severe TBI that occurred 12 to 63 months previously had poorer communication and social skills, but this was not the case for participants with a mild TBI.^14^ A Canadian study of high school children aged 13 to 20 years assessed the relationship between TBI and substance use in over 6000 participants using a cross-sectional survey design; a subsample of over 3000 participants also completed questionnaires about substance-related problems, hazardous alcohol use, and problematic cannabis use. TBI in this sample was defined as a self-reported head injury that resulted in at least a 5-minute loss of consciousness or 1 overnight hospital stay; this was correlated with concurrent items relating to medically treated injuries, which indicated that participants with a history of TBI had an average 2-fold increase in substance use in the past 12 months (adjusted odds ratios ranged from 1.87 for binge drinking to 3.77 for methamphetamine use). In the subsample assessed for substance use problems, those with a TBI history were at increased risk for problems relating to alcohol, cannabis use as well as substance-related risks as measured by the CRAFFT Screening Tool (CRAFFT is a mnemonic acronym composed of key words in each item: Car, Relax, Alone, Forget, Friends, and Trouble). However, the study did not provide information on participant age at the time or severity of the injury.^15^

A brief review of the literature makes it apparent that the differences in categorizing childhood TBI can lead to substantially different findings. We therefore attempted a systematic review of the TBI literature to develop a clearer picture of the relationship between childhood TBI and risk behavior in adolescence. Risk behavior was defined as any use of alcohol, tobacco or illicit substances, behavioral issues of conduct, or involvement in criminal activity. The review was exploratory in nature with the aims of clarifying any relationship that exists and highlighting any patterns of association such as the role of age at TBI event.

## METHODS

### Literature search

The review was carried out according to the PRISMA guidelines (www.prisma-statment.org). Electronic databases (PubMed and Web of Science) were searched until the end of March 2015 to identify English-language studies exploring the association between childhood traumatic brain injury and risk behavior in adolescence and young adulthood. The following search terms were used: ((((child*) OR (pediatric)) AND (traumatic brain or brain or head injury)) AND ((adolescen*) AND ((psychosocial or antisocial or conduct*) OR (substance ??use))) NOT (animal) NOT (adult))). At the first stage of the filtering process, titles were excluded if there was no mention of TBI or head injury; abstracts were excluded if the outcomes clearly did not relate to the risk behaviors. Following exclusion of irrelevant articles based on title and abstract, the remaining studies were screened and their references were hand-checked for any additional suitable articles.

Studies were included if they detailed (1) original research, (2) were written in English, (3) used a case control or longitudinal design, (4) reported the TBI event to have occurred between birth and 13 years of age, and (5) assessed the outcome over 13 years. Review articles, intervention studies, and reports of non-impact-related brain damage (eg, stroke or brain tumor) were excluded from the review. The cut-off age of 13 years was chosen to differentiate between childhood and adolescence as well as to ensure the outcome behaviors were being adequately measured; for example, it is uncommon for substance use to be assessed before this age.

Data were extracted on the location and design of the study, the age of the participants at injury and assessment, the identification, definition and classification of TBI, the measures used to assess outcomes, and any covariates considered in analysis. All stages of the review were conducted by EK; a 10% check carried out by MC indicated that no studies were excluded by EK that should have been included.

## RESULTS

### Characterization of studies

**Figure 1.**
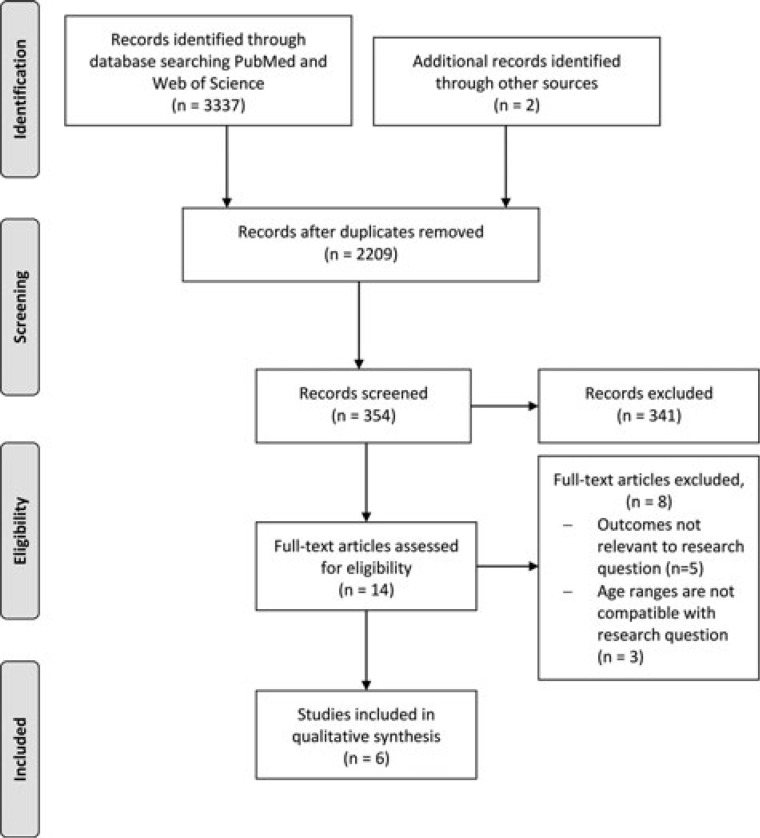
PRISMA flow diagram.

The initial search yielded 2209 articles, excluding duplicates. Fourteen journal articles were chosen for full text review, following which 8 were excluded for reasons shown in Figure 1. Six journal articles were reviewed, which were based on 4 separate studies. Two articles were from a New Zealand longitudinal study, 2 were based on an Australian longitudinal study, whereas the other 2 were from the United Kingdom and Finland. Full data extraction information can be seen in the supplementary material (see the Supplemental Digital Content, available at: http://links.lww.com/JHTR/A200).

### Summary of results

This section describes the findings of each study based on study design. Articles from the same country and cohort will be summarized in the same section. Figures relating to the study findings are presented in Table 1 because of inconsistencies in reporting across the 6 studies. Excluded studies are shown in Table 2.

**TABLE 1:** Included studies

Article	Country	Participants' age, y	Sample size; grouping	Outcomes
McKinlay et al^10^	New Zealand	Age at injury: 0–5 Age at assessment: 16–25	*N* = 953–1055 Inpatient mild TBI: *n* = 22 Outpatient mild TBI: *n* = 55–61 No injury: *n* = 876–972	*Substance use*: Inpatient alcohol OR 2.46, 95% CI 0.94–6.71, *P* < .10 Outpatient alcohol OR 1.54, 95% CI 0.75–3.12, *P* = n.s. Inpatient drug OR 2.85, 95% CI 1.11–7.32, *P* < .05 Outpatient drug OR 1.24, 95% CI 0.60–1.28, *P* = n.s. *Behavior:* Inpatient arrests IRR 4.33, 95% CI 2.55–7.34, *P* < .01 Outpatient arrests IRR 1.36, 95% CI 0.86–2.13, *P* = n.s. Inpatient property offenses IRR 2.24, 95% CI 1.42–3.52, *P* < .01 Outpatient property offenses IRR 1.35, 95% CI 0.99–1.84, *P* < .10 Inpatient violent offenses IRR 2.72, 95% CI 1.74–4.26, *P* < .01 Outpatient violent offense IRR 1.47, 95% CI 1.08–1.99, *P* < .05
McKinlay et al^12^	New Zealand	Age at injury: 0–5 Age at assessment: 14–16	*N* = 915 Inpatient mild TBI: *n* = 19 Outpatient mild TBI: *n* = 57 No injury: *n* = 839	*Substance use:* Inpatient OR 3.1, 95% CI 1.1–8.5, *P* < .05 *Behavior:* Inpatient conduct disorder/oppositional defiant disorder OR 4.9 (1.8–13.4), *P* < .01
Rosema et al^19^	Australia	Age at injury: 1–8 Age at assessment: 17–23	*N* = 104 Mild TBI: *n* = 13 Moderate TBI: *n* = 40 Severe TBI: *n* = 22 No TBI: *n* = 29	*Behavior:* Externalizing behavior, *P* = .67
Rosema et al^18^	Australia	Age at injury: 1–8 Age at assessment: mean 21.47	*N* = 54 TBI: *n* = 36 No TBI: *n* = 18	*Behavior:* Externalizing behavior, *P* = .57 Aggression, *P* = .36 Rule-breaking behavior, *P* = .46
Tonks et al^13^	United Kingdom	Age at injury: 3.7 before assessment Age at assessment: 10–16	*N* = 81 TBI: *n* = 14 No TBI: *n* = 67	*Behavior:* Conduct problems, *P* < .01 Peer problems, *P* < .01
Winqvist et al^17^	Finland	Age at injury: 0–4 Age at assessment: 0–14	*N* = 176 TBI: *n* = 176 No TBI: *n* = 10 105	*Substance use:* Drinking to intoxication, *P* < .01

Abbreviations: CI, confidence interval; IRR, increased relative risk; OR, odds ratio; TBI, traumatic brain injury.

**TABLE 2:** Excluded studies

Article	Country	Reason for exclusion
Anderson et al^32^	Australia	Outcomes not relevant
DeMatteo et al^33^	USA	Age range at injury too wide
Donders and Strom^34^	USA	Outcomes not relevant
Green et al^35^	Australia	Outcomes not relevant
McKinlay et al^11^	New Zealand	Age at outcome too young
Muscara et al^36^	Australia	Outcomes not relevant
Rosema et al^37^	Australia	Outcomes not relevant
Timonen et al^38^	Finland	Age range too wide

#### Cross-sectional

Only 1 article in the current review used a cross-sectional design, the UK study by Tonks and colleagues.^13^ This study was based on a cohort of participants recruited from occupational therapy services compared with an age- and gender-matched group of controls. The parents and teachers of the children, who were aged between 10 and 16 years at the time of assessment, completed the Strengths and Difficulties Questionnaire^16^ to investigate emotional difficulties in the participants. Both parent and teacher ratings of conduct disorders, peer problems, and negative impact of behavior in the home environment for the TBI participants were higher than for no injury controls.

#### Longitudinal

The remaining 5 articles each used a longitudinal design; 3 articles utilized data from 2 separate birth cohort studies,^10^,^12^,^17^ whereas the other 2 articles used the same follow-up data from hospital admissions.^18^,^19^

Winqvist and colleagues^17^ utilized the Northern Finland 1966 Birth Cohort, which encompasses 96% of births in the northern provinces of Finland in that year for a total of 12 058 children. Participants were grouped in terms of TBI and no TBI history based on the Finnish Hospital Discharge Register up to age 14 years. The severity of the injuries was based on the *International Classification of Diseases* (*ICD*), 8th revision. At age 14 years, all participants were asked whether they had ever drunk alcohol and if so, if they had ever been drunk. Those in the TBI group were more likely to report drinking to intoxication. Factors associated with drinking to intoxication were having a mild TBI, coming from a 1-parent family, having an urban residence, and parental alcohol misuse.

Two included articles^10^,^12^ were based on the Christchurch Health and Development Study (CHDS), an epidemiological birth cohort from New Zealand, which includes 1265 births from mid-1977. Data were gathered at birth, at 4 months and at annual intervals until age 16 years and again at ages 18, 21, and 25 years. Information was garnered from a combination of self-report, parent interview, teacher questionnaire, medical records, and other official records.^20^

In both articles, the authors focused on mild TBI grouped as “inpatient TBI” and “outpatient TBI.” The former were admitted to hospital for 2 days or fewer, whereas the latter were seen by a general practitioner or at an accident and emergency department and then sent home. For the TBI to be classified as mild, there had to have been a loss of consciousness of no more than 20 minutes; posttraumatic amnesia of less than 2 hours, if present; and no neurological signs and no evidence of skull fracture and a Glasgow Coma Scale^21^ (GCS) score of more than 14. Both groups were compared with an uninjured control group in analyses.

In the first of the 2 CHDS studies,^12^ information was gathered at ages 14 to 16 years on conduct disorder/oppositional defiant disorder (CD/ODD) and alcohol or illicit substance use/dependence using mother and self-report scales. Children who experienced an inpatient TBI between the ages of birth and 5 years had an increased likelihood of a CD/ODD *Diagnostic and Statistical Manual of Mental Disorders,* Third Edition Revised (*DSM-III-R*) diagnosis; this remained evident when maternal punitiveness at age 3 and family adverse life events were adjusted for. Likewise, inpatient TBI increased the odds of having a diagnosis of alcohol or illicit substance use/dependence at age 14 to 16 years, which remained once covariates were adjusted for.

In a later study,^10^ data were collated from self-report measures concerning alcohol dependence, drug dependence, number of arrests, property offenses, and violent offenses between the ages of 16 and 25 years. Analyses also adjusted for the individual's gender, family socioeconomic status at the child's birth, early behavior problems, and parental substance abuse/dependence. Experiencing an inpatient TBI between birth and age 5 years increased the likelihood of alcohol dependence and drug dependence. Inpatient TBI also increased the number of arrests, property offenses and violent offenses. The outpatient TBI group had an increased risk of violent offending. However, when alcohol and drug dependence were added as covariates, the increased risk of arrests, property offenses, and violent offenses were no longer supported in either group injured before age 5 years.

Participants for the remaining 2 articles were recruited from hospital admissions to the Royal Children's Hospital in Melbourne.^18^,^19^ The GCS^21^ was used to classify the severity of the injury, and a control group of uninjured children was selected from preschools and childcare centers. The participants were aged between 1 year and 7 years 11 months at the time of the injury, and both studies explored outcomes 16 years after the event.

The Adult Self-Report^22^ was used to explore externalizing behavior problems, in the first study comparing participants who had experienced a TBI to those who had not.^18^ No differences were found between the groups on self-reports of overall externalizing behavior, aggression, or rule-breaking behavior.

In another study of the same cohort,^19^ the Adult Behavior Checklist^22^ (completed by parents) revealed no differences in externalizing symptoms, between mild TBI, moderate TBI, severe TBI, and no TBI groups.

### Quality of evidence

All of the included studies were observational and therefore initially rated as having low quality of evidence based on GRADE criteria (http://www.gradeworkinggroup.org/).

#### Cross-sectional

The quality of evidence for the study by Tonks and colleagues^13^ was downgraded to very low, as there was no consideration of confounding and no effect sizes were reported. Nevertheless, participants were recruited appropriately and controls were matched for age and gender.

#### Longitudinal

The study by Winqvist and colleagues^17^ had a low quality of evidence. There was good consideration of confounding and a moderate effect size with a reasonable confidence interval. The effect size was not large enough to increase the overall quality of evidence. Strengths of this study include the large sample of participants with TBI identified from a hospital register with appropriate uninjured controls.

The McKinlay and colleagues^10^,^12^ studies had a low quality of evidence. The consideration of confounding was very good, although the confidence intervals were too wide to increase the quality to moderate. The large sample size and inclusion of an uninjured matched control group were strengths.

The Rosema and colleagues^18^,^19^ study had a very low quality of evidence. There were no effect sizes or confidence intervals reported. In one article there was no consideration of confounding, whereas in the other socioeconomic status was included as the only covariate. The sample size was small, particularly for the control groups.

## DISCUSSION

The aim of this review was to explore any association between childhood TBI and risk behavior in adolescence and young adulthood. Six articles based on 4 studies were identified: 2 birth cohort studies, 1 longitudinal follow-up study, and 1 cross-sectional study. Five articles assessed problematic behavior as an outcome of early life TBI, whereas substance use was an outcome in 3 articles. All studies compared participants with a history of TBI to participants without a TBI. In all 3 articles exploring substance use, a positive relationship was found between TBI and substance use.^10^,^12^,^17^ Findings relating to behavioral issues were less consistent across the 5 articles; the TBI groups in 3 of the articles had poorer behavioral outcomes,^10^,^12^,^13^ whereas there were no differences between groups in the remaining 2 articles.^18^,^19^

The quality of evidence for all 4 studies ranged from low to very low, in part due to the observational design of the studies. The cross-sectional study^13^ and the prospective longitudinal study^18^,^19^ were downgraded to a very low quality of evidence as neither study adequately controlled for plausible confounding factors, and both had relatively small sample sizes. In addition, the study by Tonks and colleagues^13^ reported neither effect size estimates nor confidence intervals. Both birth cohort studies^10^,^12^,^17^ were rated as providing low quality of evidence; notably, plausible confounding was taken into consideration and the sample sizes were large. There was some indication of a dose-response relationship between injury severity and the outcomes of interest, but this differed between the 2 studies; Winqvist and colleagues^17^ found an association with mild TBI and drinking to intoxication, whereas McKinlay and colleagues^10^,^12^ found that a certain threshold of mild TBI was necessary for an association to be seen. The effect sizes and confidence intervals were not of great enough magnitude in either study to increase the quality of evidence rating from low to moderate.

A considerable strength of the included articles is the use of medical records to identify and classify TBI, and also the consistency of the use of the GCS across 3 of the 4 included studies. (The GCS was unavailable when injury was assessed in the Northern Finland 1966 Birth Cohort.^17^) However, the TBI groups were variously formed based on severity in terms of mild versus moderate-to-severe,^17^,^19^ severity of a mild TBI,^10^,^12^ or the presence of a TBI,^13^,^18^ which makes comparison more difficult. In addition, there is some question about the sensitivity of the GCS to measure milder injuries; for example, Rees argued that a maximum score of 15 does not help in determining whether a brain injury has occurred. Three articles found relationships between risk behavior and mild TBI; however, the severity was classified differently. Winqvist and colleagues^17^ classed participants as having a mild TBI based on *ICD* 8^th^ Revision codes corresponding to concussion and skull fractures; however, it is unclear whether the inclusion of skull fractures could be more in keeping with the “complicated mild” level of severity put forward by Williams and colleagues^24^ who found neurobehavioral outcome at 6 months was comparable to that for persons with moderate injury when the mild TBI included a depressed skull fracture or brain lesion. Conversely, McKinlay and colleagues^10^,^12^ excluded participants from the mild TBI group if there was evidence of a skull fracture, and they used loss of consciousness of less than 20 minutes as one signifier of a mild injury. This length of time is in keeping with a recent report for the Children's Commission where a mild injury was defined as a loss of consciousness of between 10 and 20 minutes^25^; however the American Congress of Rehabilitation Medicine definition suggests that a loss of consciousness of up to 30 minutes still signifies a mild TBI.^26^ There is a need for clarification and harmonization across studies. One important caveat is that relying on medical records alone may misrepresent the prevalence of TBI; higher rates of self-reported TBI compared with rates obtained through medical records suggest that not all those who incur a TBI will present to medical services.^25^ This may be particularly pertinent if, for example, the TBI was sustained in the context of illegal activity.

The control groups in all included studies were age-matched participants without a history of TBI. It has been argued that an additional trauma group should be included in studies of TBI to control for factors associated with injury that may be poorly measured.^27^ Rees^28^ reviewed 5 articles that assessed persistent postconcussive syndrome in mild TBI and in non-brain-related injuries and reported comparable outcomes between both groups. In a study of postinjury substance use among participants with a TBI and a spinal cord injury, Kolakowsky-Hayner and colleagues^29^ reported no differences in drinking patterns but higher rates of illicit drug use in participants with a spinal cord injury than those with a TBI. Satz^30^ has recommended that in order to confirm a head injury rather than a general injury effect, a difference between a head injury and other injury group as well as a difference between a head injury and no injury group must be observed. To control for injury factors such as pain experience or posttraumatic stress,^28^ future research should aim to include an extracranial injury group alongside an uninjured control group to act as a negative control. Negative control design is employed to uncover potentially unmeasured confounding or bias by comparing the main analysis of interest to a second analysis between the negative control exposure and main outcome. If there is an association of larger magnitude between the exposure of interest and the outcome, then it adds to the strength of evidence for a causal association. The negative control chosen must have no plausible biological mechanism for the association with the outcome of interest and have a similar confounding structure to the outcome of interest.^31^

The evidence presented in this review indicates that the associations between childhood TBI and later risk behaviors are not yet understood. However, there are some limitations to this review. First, the literature search yielded a rather small set of articles based on 4 unique participant samples. One possibility is that the exclusion of non-English language publications may have resulted in some relevant articles being missed. No librarian was involved in the search strategy, which may have been beneficial. However, the low number of studies may simply indicate a paucity of research on the long-term effects of childhood TBI on risk behavior. Second, it was not possible to carry out a quantitative synthesis (ie, meta-analysis) on the results because of the variety of outcomes assessed and the differences in TBI groupings. For example, within 3 articles, substance use was measured in terms of drinking alcohol to intoxication,^17^ through survey questions^12^ or by use of the Composite International Diagnostic Interview.^10^ Third, although the quality of evidence for observational studies is rated as low by the GRADE approach, 2 included studies were downgraded to very low. This makes it more difficult to draw firm conclusions and could be avoided in future by adjusting for all potential confounders and clearly reporting effect sizes and confidence intervals.

Although the articles reviewed here provide some support for a link between early life TBI and later risk behavior, particularly substance use,^10^,^12^,^17^ much more research needs to be undertaken before any clear conclusions can be drawn. The quality of evidence in the included studies was low to very low; effect sizes and confidence intervals should be clearly reported and analyses on the effect of injury severity should be carried out. We suggest that future longitudinal research build on these articles by (1) continued use of medical records combined with self-report measures for the identification and classification of severity of TBI, and (2) by controlling for general injury effects through use of a control group with non-brain-related injury and potentially through the use of neuroimaging techniques.
